# Training changes processing of speech cues in older adults with hearing loss

**DOI:** 10.3389/fnsys.2013.00097

**Published:** 2013-11-28

**Authors:** Samira Anderson, Travis White-Schwoch, Hee Jae Choi, Nina Kraus

**Affiliations:** ^1^Auditory Neuroscience Laboratory, Department of Communication Sciences and Disorders, Northwestern UniversityEvanston, IL, USA; ^2^Department of Communication Sciences, Northwestern UniversityEvanston, IL, USA; ^3^Institute for Neuroscience, Northwestern UniversityEvanston, IL, USA; ^4^Department of Neurobiology and Physiology, Northwestern UniversityEvanston, IL, USA; ^5^Department of Otolaryngology, Northwestern UniversityEvanston, IL, USA

**Keywords:** auditory plasticity, speech envelope, aging, hearing loss, speech perception, temporal fine structure, temporal coding

## Abstract

Aging results in a loss of sensory function, and the effects of hearing impairment can be especially devastating due to reduced communication ability. Older adults with hearing loss report that speech, especially in noisy backgrounds, is uncomfortably loud yet unclear. Hearing loss results in an unbalanced neural representation of speech: the slowly-varying envelope is enhanced, dominating representation in the auditory pathway and perceptual salience at the cost of the rapidly-varying fine structure. We hypothesized that older adults with hearing loss can be trained to compensate for these changes in central auditory processing through directed attention to behaviorally-relevant speech sounds. To that end, we evaluated the effects of auditory-cognitive training in older adults (ages 55–79) with normal hearing and hearing loss. After training, the auditory training group with hearing loss experienced a reduction in the neural representation of the speech envelope presented in noise, approaching levels observed in normal hearing older adults. No changes were noted in the control group. Importantly, changes in speech processing were accompanied by improvements in speech perception. Thus, central processing deficits associated with hearing loss may be partially remediated with training, resulting in real-life benefits for everyday communication.

## Introduction

Hearing loss is associated with reduced quality of life in older adults, affecting social and emotional well-being (Heine and Browning, 2002). The effects of hearing loss are especially noticeable in noisy backgrounds with multiple talkers (Dubno et al., 1984; Jin and Nelson, 2010). Many factors contribute to age-related deficits in speech-in-noise perception, including cochlear pathology (Dubno et al., 1984), impairments in central auditory processing (Phillips et al., 2000), and decreased cognitive resources (Pichora-Fuller, 2003; Wingfield et al., 2005; Peelle et al., 2010). Perceptual and neurophysiologic studies have demonstrated that central auditory processing is compromised by aging and hearing loss (Gordon-Salant et al., 2006; Clinard et al., 2010; Lister et al., 2011; Anderson et al., 2012). Hearing aid amplification improves audibility but cannot restore central auditory function (Tremblay et al., 2003). Training-driven neuroplasticity, which can partially reverse the effects of age-related deficits in temporal resolution in humans (Anderson et al., 2013c) and animals (de Villers-Sidani et al., 2010), may provide a means for treating abnormal central function processing associated with hearing loss.

One chief barrier to the wider use of amplification has been that hearing aids often make speech louder without improving clarity, especially in background noise (Johnson and Dillon, 2011). This phenomenon may arise from the effects of hearing loss on central processing of speech. Two acoustic aspects of speech, the slowly varying temporal envelope and the rapidly varying temporal fine structure (TFS), have been extensively studied (Ardoint et al., 2010; Hopkins and Moore, 2011). Although the envelope appears to be the dominant cue for understanding speech in quiet and in steady-state noise, both the envelope and TFS may play a role when listening to speech in fluctuating noise (Shannon et al., 1995; Moore, 2008). However, the role of TFS for understanding speech in fluctuating noise remains an area of debate (Oxenham and Simonson, 2009). In an animal model, sensorineural hearing loss is associated with enhanced envelope coding in the auditory nerve (Kale and Heinz, 2010) and midbrain (Anderson et al., 2013a). Perceptual studies also suggest exaggerated encoding of the envelope in humans with unilateral hearing loss (Moore et al., 1996) and potentially reduced ability to use TFS cues (Lorenzi et al., 2006). These effects might explain the hearing impaired listener's complaint that speech is loud yet unclear (Johnson and Dillon, 2011).

Because hearing aid amplification primarily addresses loss of audibility associated with cochlear pathology, there is a need to develop novel methods for counteracting the deleterious effects of hearing loss on central processing. Here, we assessed whether auditory-based cognitive training can be used to partially restore the imbalance of speech cue representation associated with exaggerated envelope encoding. Since both TFS (Sheft et al., 2008) and envelope (Swaminathan and Heinz, 2012) cues play an important role in consonant perception, we hypothesized that directed attention to the fast-changing consonant-vowel (CV) transition within an adaptive training paradigm results in reweighing of speech cue representation, such that the TFS becomes more salient. To test this hypothesis, we randomly assigned older adults with and without hearing loss to complete 8 weeks of computerized training. We evaluated subcortical representation of envelope and TFS cues before and after training in addition to speech perception in noise, auditory short-term memory, and auditory attention.

## Materials and methods

### Participants

We recruited 58 (35 female) participants (ages 55–79) for a study examining training for speech-in-noise processing. Pure-tone audiometric thresholds were obtained bilaterally at octave intervals 0.125–8 kHz and at 3 and 6 kHz. No left/right asymmetries or interaural click-evoked auditory brainstem response Wave V differences (≥0.2 ms) were noted. No participants had a history of neurologic conditions and all participants had normal IQs [≥85 on the WASI (Zhu and Garcia, 1999)]. Participants provided informed consent for procedures that were approved by the Northwestern Institutional Review Board and were paid for their time.

**Figure 1:**
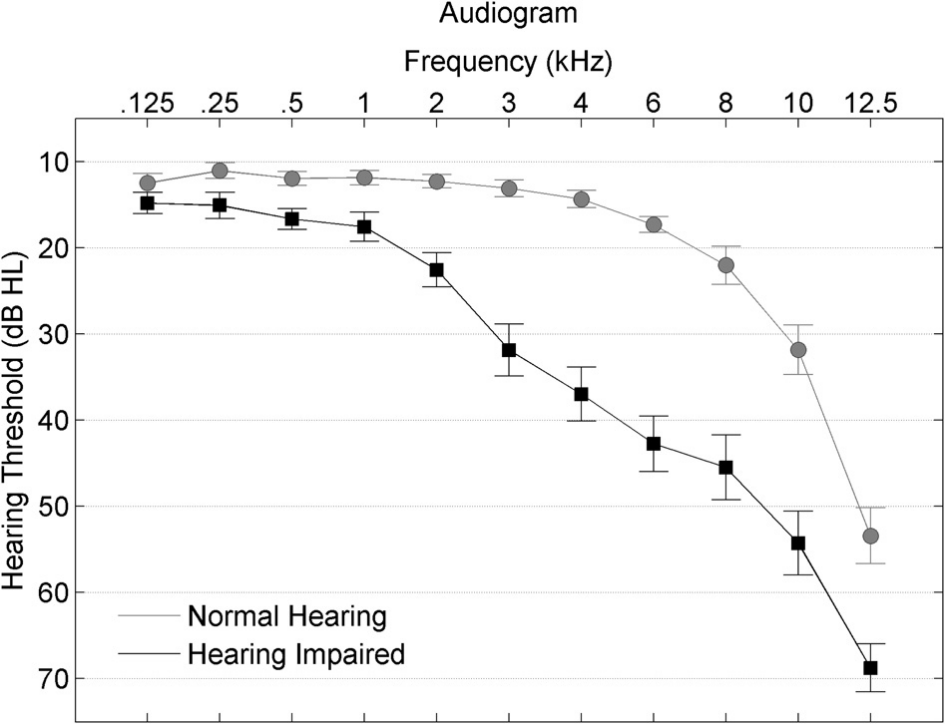
**Average hearing thresholds are plotted from 0.125–8 kHz for participants with normal hearing (gray circles) and with hearing loss (black squares), with error bars ± 1 SE**.

**Figure 2:**
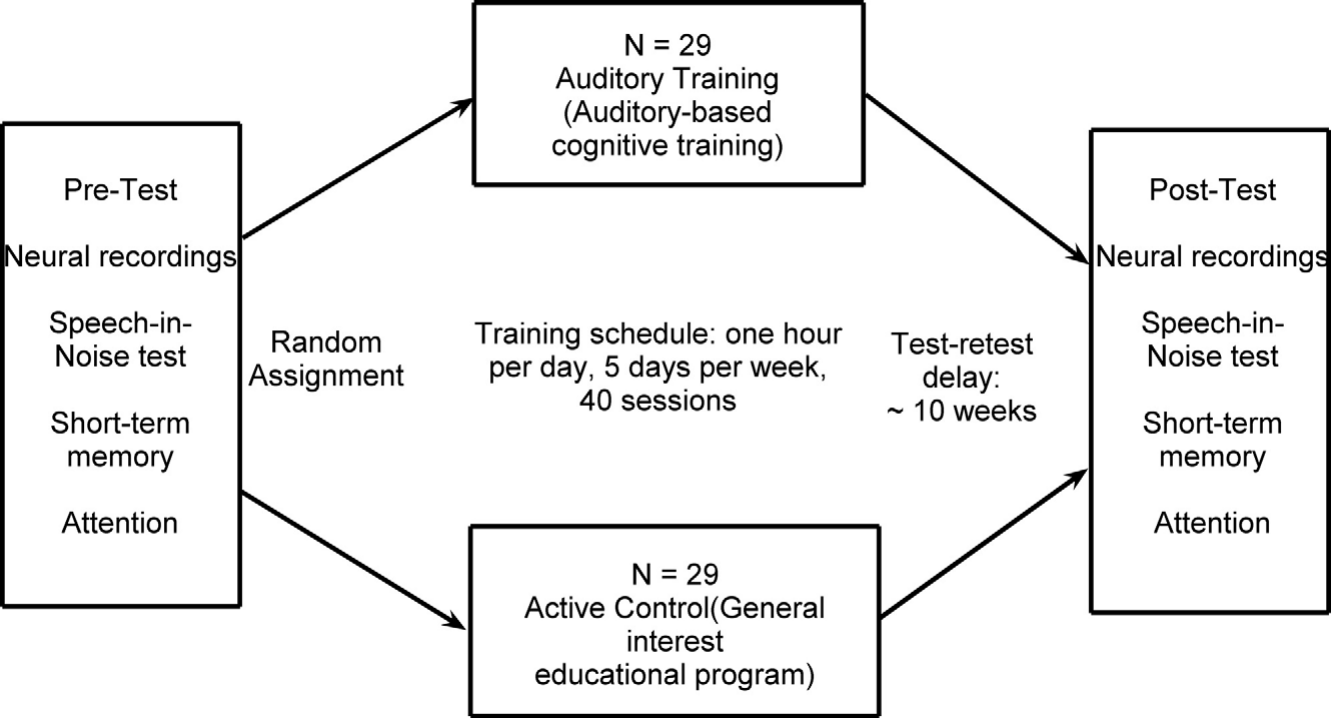
**A summary of the experimental design**.

Participants were divided into normal-hearing and hearing-impaired subgroups. The normal-hearing participants had hearing thresholds ≤25 dB HL through 6 kHz and the participants with hearing loss had hearing thresholds ≤80 dB HL through 8 kHz. See Figure 1 for average thresholds in the normal-hearing and hearing-impaired groups. Participants from both hearing groups were randomly assigned to complete either auditory-based cognitive training or active control training (Smith et al., 2009). Both involved training on an in-home computer, 1 h/day, 5 days/week, for 8 weeks. The *auditory training* group completed Brain Fitness™ cognitive training (Posit Science Corporation, San Francisco, CA) consisting of six modules designed to increase the speed and accuracy of auditory processing: (1) time-order judgments of frequency-modulated sweeps, (2) discriminating between pairs of confusable syllables, (3) recognizing sequences of confusable syllables and words, (4) matching pairs of confusable syllables and words, (5) implementing sequences of commands, and (6) answering questions from stories (see Smith et al., 2009 for details). In the first module, an adaptively decreasing inter-stimulus interval challenges processing speed. In subsequent modules, focused attention is directed to adaptively expanding and contracting CV transitions. The overall goal of the training is to improve sensory function, based on the idea that an increase in the quality of neural information flowing through peripheral and central sensory systems may lead to better cognitive function (Schneider and Pichora-Fuller, 2000). Completion of training was verified through automated online logs. The *active control* group watched educational DVDs and completed multiple-choice questions about the content. See Figure 2 for a schematic of the experimental design. Training groups (and hearing subgroups) were matched for sex, age, hearing, click-evoked wave V brainstem latency, IQ, and test-retest intervals (Table 1).

**Table 1:** **Means and *SD*s are provided for age, IQ, and hearing (PTA dB HL; average hearing threshold 0.5–4 kHz) for all participants in the *Auditory Training* and *Active Control* groups and the subgroups of normal hearing and hearing impaired**.

**Mean (*SD*)**	**Auditory training**			**Active control**		
	**Total**	**Normal hearing**	**Hearing impaired**	**Total**	**Normal hearing**	**Hearing impaired**
	**(*N*** = **29)**	**(*N*** = **14)**	**(*N*** = **15)**	**(*N*** = **29)**	**(*N*** = **14)**	**(*N*** = **15)**
Age	64.11 (5.78)	60.86 (3.74)	67.36 (5.72)	64.07 (5.22)	62.86 (3.34)	65.29 (6.50)
IQ	118.43 (12.15)	117.00 (12.30)	119.36 (12.43)	120.00 (14.23)	118.36 (14.28)	122.56 (14.61)
Hearing (dB HL)	20.64 (11.29)	11.36 (3.18)	28.25 (8.19)	18.3 (7.83)	13.43 (3.20)	22.87 (8.19)
Sex (males)	9	5	4	14	8	6

The groups are matched on all listed variables (all p's > 0.10).

### Behavioral assessments

#### Perceptual—speech perception in noise

The QuickSIN is a non-adaptive measure of speech perception in noise. Sentences are presented in a background of four-talker babble and the SNR decreases by 5 dB for each sentence. Participants receive a point for correctly repeating target words. The total number of points is subtracted from 25.5 to arrive at a final SNR loss [or the increase in SNR required to achieve 50% correct performance compared to normal performance of 0 dB SNR; see Killion et al. (2004)].

#### Cognitive

Two subtests of the Woodcock-Johnson III Cognitive Test Battery (Woodcock et al., 2001) were used to obtain an age-normed cluster score for auditory short-term memory: Numbers Reversed and Memory for Words. The attention score was based on the sustained index of overall attention (visual and auditory) on the IVA+: Integrated Visual and Auditory Continuous Performance Test (BrainTrain, North Chesterfield, VA). Due to equipment malfunction, we are missing attention data from 3 participants in the *auditory training* group and 1 participant in the *active control* group.

### Electrophysiology

#### Stimulus

**Figure 3:**
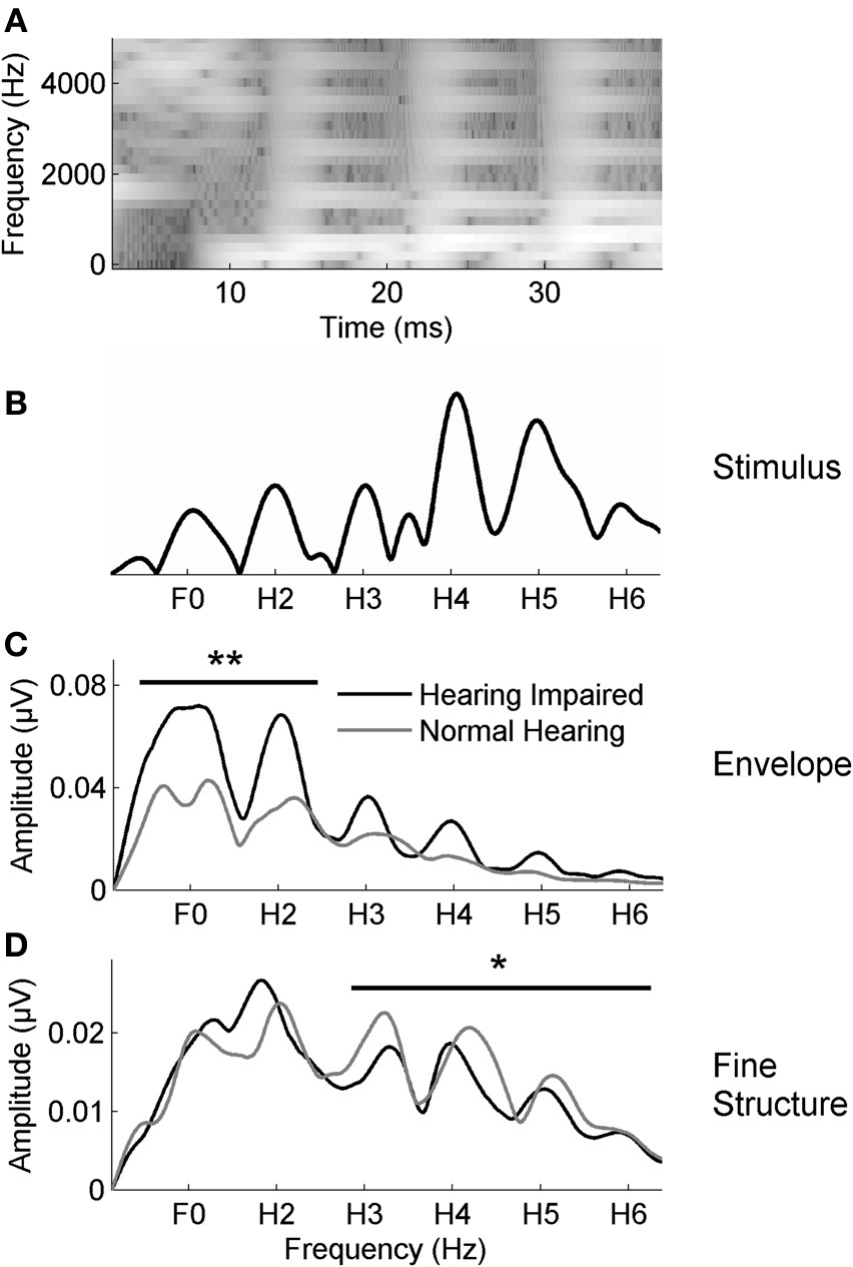
**(A)** The spectrogram of the 40-ms syllable [da]. Fast Fourier transforms were calculated from 20 to 42 ms for the stimulus **(B)** and in responses to the envelope **(C)** and the TFS **(D)** in noise. The average responses for the participants with normal hearing (gray) and with hearing loss (black) are displayed. The group with hearing loss has higher amplitudes in the envelope-dominated low frequencies (F_0_–H_2_) in noise relative to the group with normal hearing **(C)**; conversely, the group with normal hearing has higher amplitudes in the fine structure dominated higher frequencies (H_3_–H_6_) than the group with hearing loss **(D)**. ^*^p < 0.05, ^**^p < 0.01.

We duplicated the electrophysiologic methods previously used to document enhanced envelope coding in older adults with hearing loss (Anderson et al., 2013a), using the protocol that produced the greatest hearing loss effects. The stimulus was a 40-ms syllable [da] synthesized in a Klatt-based synthesizer (Klatt, 1980). It began with a 5-ms onset burst followed by a CV transition and was perceived as a full CV syllable although it lacked a steady-state vowel. After the initial onset burst, the fundamental frequency (F_0_) of the stimulus rose linearly from 103 to 125 Hz while the formants shifted as follows: F_1_: 220 → 720 Hz; F_2_: 1700 → 1240 Hz; F_3_: 2580 → 2500 Hz. The fourth (3600 Hz) and fifth (4500 Hz) formants remained constant for the duration of the stimulus. A spectrogram and Fourier transform of the stimulus waveform are presented in Figures 3A,B, respectively.

To partially equate for the effects of hearing loss, the [da] stimulus was individually amplified based on the National Acoustics Laboratory-Revised algorithm (NAL-R; Byrne and Dillon, 1986) using a custom program in MATLAB (The MathWorks, Inc., Natick, MA). The amplified presentation level did not exceed 90 dB SPL or the participants' loudness discomfort thresholds. We have previously found that the amplification procedure improves morphology without distorting spectral components of the response (Anderson et al., 2013b). In addition, although we found enhanced encoding in brainstem responses to both amplified and unamplified stimuli, the differences were clearest for the amplified stimuli (Anderson et al., 2013a); therefore, we expected to see the greatest treatment effects in response to the amplified stimuli. Repeated hearing tests verified that there were no changes in hearing after 8 weeks and therefore all participants received the same amplified stimulus before and after training.

#### Recording

The [da] was presented binaurally in pink noise at +10 dB SNR. Stimuli were presented at 80 dB SPL for normal hearing listeners and amplified stimuli for participants with hearing loss were presented at an intensity of up to 90 dB SPL, with most stimuli in the range of 80–83 dB SPL. Stimuli were presented via the Bio-Logic Navigator Pro System (Natus Medical, Inc., Mundelein, IL) at a rate of 10.9 Hz (inter-stimulus interval of 52 ms) through electromagnetically shielded insert earphones (ER-3A, Etymotic Research, Elk Grove Village, IL). A vertical montage of four Ag-AgCl electrodes (Cz active, Fpz ground, earlobe references) was used with all contact impedances <5 kΩ. A criterion of ± 23 μV was used for online artifact rejection. Two blocks of 3000 artifact-free sweeps were collected in each condition for each participant and averaged using an 85.3-ms window, including a 15.8-ms prestimulus period. Responses were sampled at 12 kHz and were online bandpass filtered from 100 to 2000 Hz (Butterworth filter, 12 dB/octave, zero phase-shift) to minimize disruption by the low-frequency cortical response and to sample energy up to the phase-locking limits of the brainstem (Liu et al., 2006).

The [da] was presented in alternating polarities, allowing for the creation of responses comprised of both the sum and the difference of the two polarities (Campbell et al., 2012). When creating the summed frequency following response (FFR), the non-inverting envelope component of the response is enhanced while the inverting TFS component is minimized; conversely, for the subtracted responses the inverting TFS component is enhanced while the non-inverting envelope component is substantially reduced (Aiken and Picton, 2008). While this analysis is a somewhat different operationalization of envelope and TFS representation than is used in some other studies, the selection of measures of envelope and TFS representation is driven by the model organization and site of interest (Shamma and Lorenzi, 2013), and adding/subtracted brainstem responses to alternating polarities is a typical method used in analyzing human FFRs (Aiken and Picton, 2008; Gockel et al., 2011; Anderson et al., 2013a). Nevertheless, we note that the relationship between envelope/TFS representation reflected in the FFR, perceptual measures, and auditory nerve coding remains an avenue for future research. Spectral amplitudes were calculated using fast Fourier transforms (FFTs) over 60 Hz bins around the frequencies of interest, which included the fundamental frequency (F_0_) and its integer harmonics. The time region chosen for this calculation was 20–42 ms, corresponding to the most periodic time region of the FFR.

### Statistical analysis

A Multivariate Analysis of Variance (MANOVA) was used to assess hearing group differences in the envelope and TFS. The F_0_ and H_2_ amplitudes from the added polarities (henceforth F_0ADD_–H_2ADD_) were entered as dependent variables in the MANOVA to represent the envelope, as these lower frequency spectral peaks dominate the envelope-following FFR. The H_3_–H_6_ amplitudes from the subtracted polarities (H_3SUB_–H_6SUB_) were entered as dependent variables to represent the TFS, which is more prominent in relatively higher frequencies in the FFR. A repeated measures ANOVA was used to compare envelope-dominated (F_0ADD_–H_2ADD_) and TFS-dominated (H_3SUB_–H_6SUB_) frequency encoding, QuickSIN SNR scores, and memory and attention measures before and after training.

## Results

### Effects of hearing loss on encoding speech envelope/TFS

The combined participants with hearing loss from both groups had greater representation of the envelope (F_0ADD_ and H_2ADD_) than participants with normal hearing [*F*_(1, 57)_ = 7.218, *p* = 0.002] (Figure 3C), replicating a previous study demonstrating greater subcortical representation of the envelope in response to speech in noise in older adults with hearing loss (Anderson et al., 2013a). In contrast to the Anderson et al. study (2013a), however, we also found reduced representation of the TFS (H_3SUB_–H_6SUB_) in the older adults with hearing loss [*F*_(1, 57)_ = 3.066, *p* = 0.024] (Figure 3D). The current study comprised 58 participants vs. the 30 participants in the previous study; therefore, the new finding of significant differences in TFS representation in the current study may be attributed to increased power and is in fact consistent with Henry and Heinz (2012), who found a reduction in TFS coding in noise in auditory nerve fibers of chinchillas with noise-induced hearing loss.

### Training: neurophysiological changes

**Figure 4:**
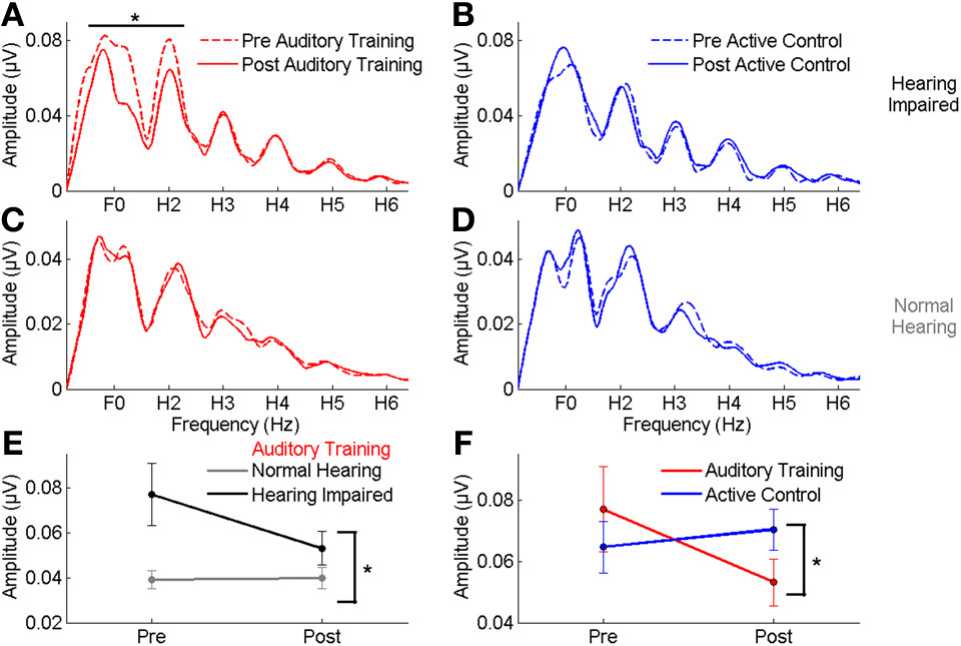
**(A,B)** A comparison of pre- (dotted lines) and post-training responses (solid lines) to speech in noise to the envelope (F_0_–H_2_) in the *auditory training* (red) and *active control* (blue) groups with hearing loss, demonstrating a significant reduction in response to the envelope in the *auditory training* group. **(C,D)** No reduction was seen in response to the envelope in either group with normal hearing. **(E)** A significant hearing × session interaction was noted in the *auditory training* group, demonstrating that the change was specific to the participants with hearing loss. **(F)** A significant group × session interaction in the groups with hearing loss indicating a reduction in the representation of the F_0_ in the *auditory training* group only. ^*^p < 0.05. Error bars: ± 1 SE.

**Figure 5:**
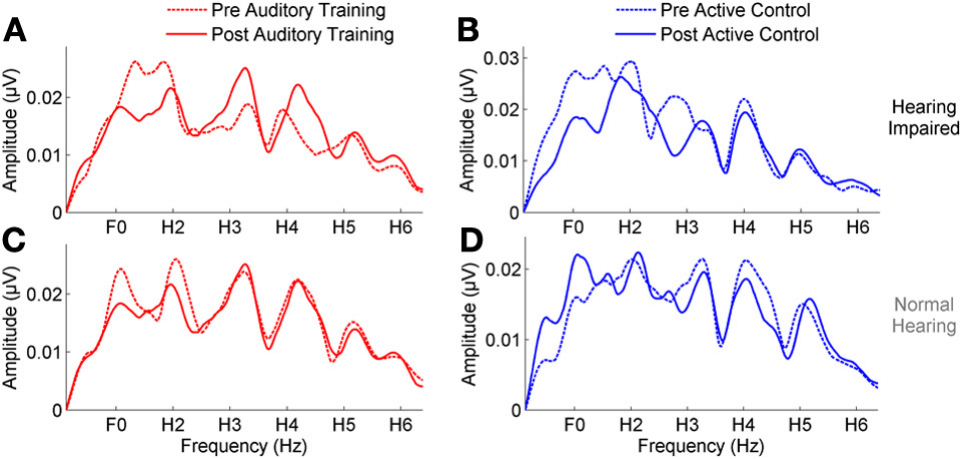
**(A,B)** A comparison of pre- (dotted lines) and post-training responses (solid lines) to speech in noise to the fine structure (H_3_–H_6_) in the *auditory training* (red) and *active control* (blue) groups with hearing loss, demonstrating no change in either group. **(C,D)** No change was seen in response to the fine structure in either group with normal hearing.

**Figure 6:**
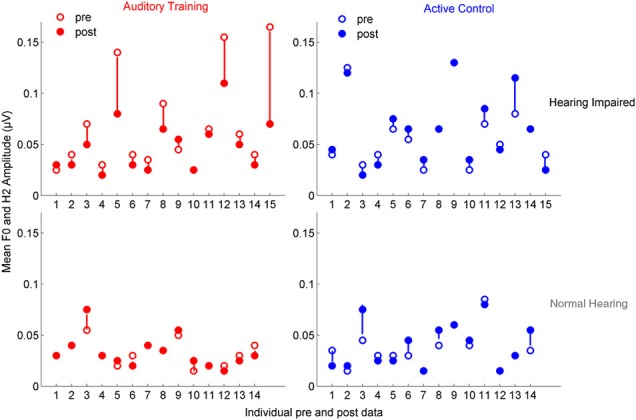
**Mean F_0ADD_ and H_2ADD_ amplitudes are displayed for individual pre- (open circles) and post-data (closed circles) for the auditory training (red) and active control (blue) groups**. Visual observation of the data reveals that there is greater pre-training variability in both groups with hearing loss and in the degree of change in the auditory training group with hearing loss.

**Figure 7:**
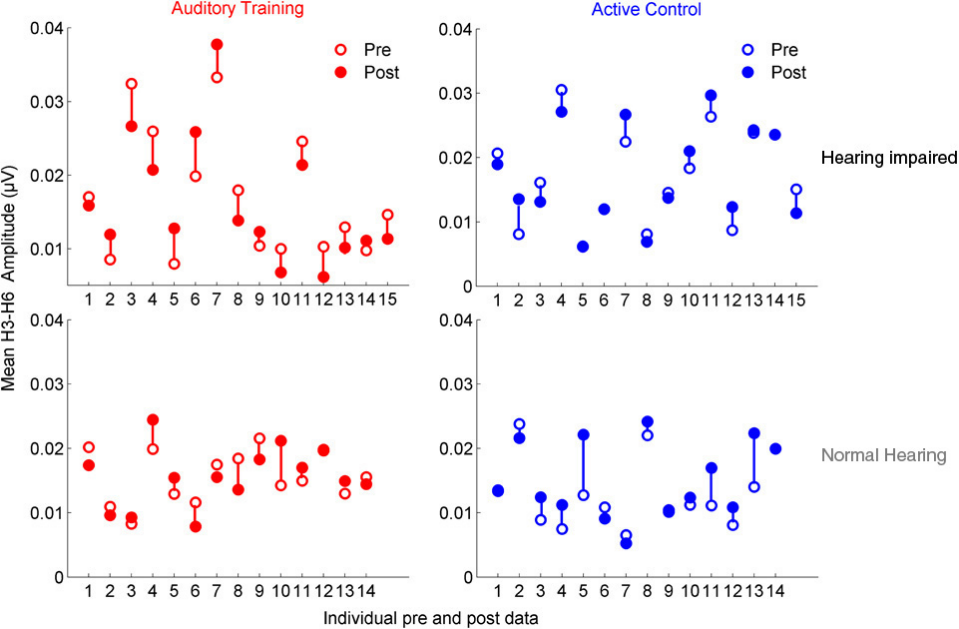
**Mean H_3SUB_ and H_6SUB_ amplitudes are displayed for individual pre- (open circles) and post-data (closed circles) for the auditory training (red) and active control (blue) groups**. Similar to the data for the envelope, the data demonstrates greater variability in both groups with hearing loss for pre-test data, but there is no systematic change with training as was found for the envelope.

In the group with hearing loss, we found a training group × test session interaction [*F*_(1, 30)_ = 4.351, *p* = 0.023], with a significant reduction in envelope encoding (F_0ADD_ to H_2ADD_) occurring within the *auditory training* group [*F*_(1, 14)_ = 3.843, *p* = 0.049] but not the *active control* group [*F*_(1, 14)_ = 0.381, *p* = 0.691] (see Figures 4A,B). We also analyzed the pre and post data of the groups with normal hearing and found no training group × test session interaction [*F*_(1, 27)_ = 0.803, *p* = 0.459] and no changes in either group (all *p*'s > 0.1) (see Figures 4C,D). Although there was no training group × hearing group × test session interaction [*F*_(1, 57)_ = 2.239, *p* = 0.117], there was a hearing group × test session interaction in the *auditory training* group [*F*_(1, 29)_ = 3.573, *p* = 0.043] that was not present in the *active control* group [*F*_(1, 29)_ = 0.136, *p* = 0.874] (see Figures 4E,F), suggesting that the auditory training effect was specific to the participants with hearing loss. Representation of the TFS (H_3SUB_–H_6SUB_) did not change for either hearing impaired or normal hearing participants of either training group (all *p*'s > 0.1) (Figure 5). Figures 6, 7 display mean F_0ADD_ and H_2ADD_ and mean H_3SUB_–H_6SUB_ amplitudes, respectively, for individual participants. It is evident from these figures that individuals with hearing loss have greater variability than individuals with normal hearing.

### Training: behavioral changes

Summary: There were significant training-induced changes in speech-in-noise perception, memory, and attention across hearing groups in the *auditory training* group. The training effects specific to hearing status varied depending on the task. The improvement in speech-in-noise performance was specific to the hearing impaired group, the memory change was found only in the normal hearing group, and attention improved in both groups. There were no corresponding changes in the *active control* group.

For speech-in-noise performance (QuickSIN), there was a significant training group × test session interaction [*F*_(1, 57) = 5.191_, *p* = 0.027], with improvement noted in the *auditory training* group [*F*_(1, 28)_ = 13.394, *p* = 0.001] but not in the *active control* group [*F*_(1, 28)_ = 1.678, *p* = 0.206]. This change was largely driven by the improvement in performance in the auditory training group with hearing loss [*F*_(1, 14)_ = 12.220, *p* = 0.004]; the improvement in the normal-hearing group was not significant [*F*_(1, 14)_ = 3.041, *p* = 0.105]. Neither hearing group in the *active control* group changed with training (all *p's > 0.1*). There was a decrease in the QuickSIN of 1.22 dB in the *auditory training* group with hearing loss—given four lists, this number is below the 1.9 dB necessary for a critical difference between conditions with an 95% confidence interval (Killion et al., 2004). However, a 1 dB decrease in SNR corresponds to approximately a 10% increase in word recognition—a difference that is likely to be noticeable to the listener (Middelweerd et al., 1990). It should also be noted that the participants with hearing loss did not have more than a mild SNR loss—less than 7 dB—so greater gains might be expected from individuals with greater deficits.

Similarly, for short-term memory there was a significant training group × test session interaction [*F*_(1, 57)_ = 6.042, *p* = 0.017], with improvements in the *auditory training* group [*F*_(1, 28)_ = 9.800, *p* = 0.004] but not the *active control* group [*F*_(1, 28)_ = 0.158, *p* = 0.694]. For memory, however, the changes were only significant in the group with normal hearing [*F*_(1, 28)_ = 7.648, *p* = 0.016] but not in the group with hearing loss [*F*_(1, 28)_ = 2.630, *p* = 0.127] and neither hearing group in the *active control* group changed (all *p*'s > 0.1).

**Figure 8:**
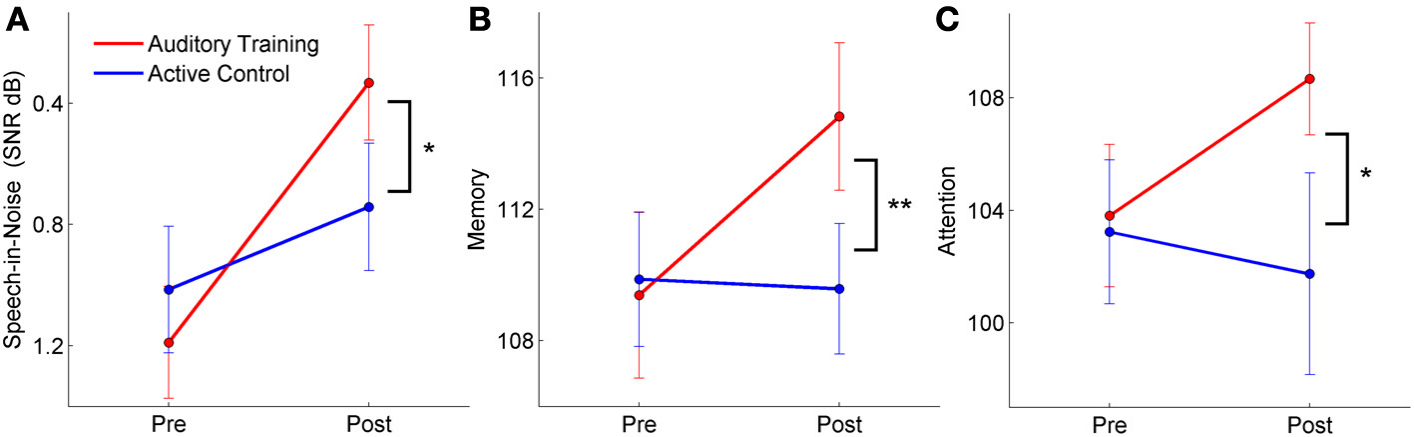
**Pre and post-training perceptual and cognitive scores for participants with both normal hearing and hearing loss (group × session interactions)**. The *auditory training* group improved in speech-in-noise perception **(A)**, memory **(B)**, and attention **(C)**, whereas there were no changes in the *active control* group. ^*^p < 0.05, ^**^p < 0.01. Error bars: ± 1 SE.

Finally, for attention, there was a significant training group × test session interaction [*F*_(1, 50)_ = 3.765, *p* = 0.043], with improvements in the *auditory training* group [*F*_(1, 25)_ = 17.941, *p* < 0.001] but not the *active control* group [*F*_(1, 24)_ = 0.623, *p* = 0.438]. In this case, there were significant improvements for both the subgroups with normal hearing [*F*_(1, 11)_ = 8.182, *p* = 0.016] and with hearing loss of the *auditory training* group [*F*_(1, 12)_ = 9.339, *p* = 0.009]. Again, there were no changes for members of either hearing subgroup of the *active control* group (all *p*'s > 0.1). See Figure 8 for interaction plots of behavioral changes. Please refer to Table 2 for means and standard deviations of pre- and post-training changes in behavioral measures for the normal hearing and hearing impaired participants of the *auditory training* and *active control*, groups.

**Table 2:** **Means and *SD*s are provided for pre- and post-test scores for speech-in-noise perception, auditory short-term memory and attention for the *Auditory Training* and *Active Control* groups, including the subgroups of participants with normal hearing and with hearing loss**.

**Mean (*SD*)**	**Session**	**Auditory training**			**Active control**		
		**Total**	**Normal hearing**	**Hearing impaired**	**Total**	**Normal hearing**	**Hearing impaired**
		**(*N*** = **29)**	**(*N*** = **14)**	**(*N*** = **15)**	**(*N*** = **29)**	**(*N*** = **14)**	**(*N*** = **15)**
Speech-in-noise perception	Pre	1.51 (1.44)	0.72 (0.82)	2.04 (1.38)	1.13 (1.16)	0.93 (1.26)	1.31 (1.07)
	Post	0.65 (1.27)	0.25 (1.08)	0.84 (1.32)	0.91 (1.22)	0.63 (1.14)	1.18 (1.27)
Memory	Pre	09.28 (14.24)	104.71 (12.77)	113.53 (14.62)	110.38 (11.65)	113.53 (14.62)	109.80 (7.72)
	Post	114.24 (13.08)	111.43 (14.32)	116.57 (11.67)	109.72 (11.34)	116.57 (11.67)	108.07 (10.87)
Attention	Pre	103.48 (14.47)	99.08 (17.44)	107.00 (10.96)	103.62 (15.48)	100.00 (16.51)	107.11 (12.27)
	Post	108.25 (11.81)	104.50 (12.38)	112.00 (10.30)	99.35 (23.61)	100.46 (24.90)	105.37 (24.24)

## Discussion

Here we show that the imbalance in neurophysiological processing of speech cues associated with hearing loss is malleable and reversible with training in older adults. The results are summarized as follows: first, compared to normal hearing individuals, older adults with hearing loss have excessively large envelope encoding of speech in noise. Second, training that targets the CV transition reduces envelope representation in individuals with hearing loss to levels in line with those of normal-hearing individuals. Finally, the training-induced changes in neurophysiology are accompanied by gains in speech-in-noise perception, attention, and short-term memory, although it should be noted that the change in speech-in-noise perception was modest, and would not be considered clinically significant (Killion et al., 2004). Nevertheless, we note that an improvement of 1 dB SNR corresponds to approximately 10% word intelligibility in noise (Middelweerd et al., 1990), so the changes may be perceived as beneficial to the listener.

Our results confirm previous findings of exaggerated representation of envelope cues in animal and human models of sensorineural hearing loss (Kale and Heinz, 2010; Henry and Heinz, 2012; Anderson et al., 2013a), providing a possible explanation for the observation that the hearing impaired listener perceives speech as loud but unclear (Jin and Nelson, 2010). At present, the mechanisms underlying this exaggerated representation in auditory brainstem are unknown; however, evidence from auditory nerve suggests a peripheral etiology arising from reduced outer hair cell compression in cases of mild to moderate hearing loss and from inner hair cell damage (steeper input-output functions) in cases of moderate to severe hearing loss (Kale and Heinz, 2010). The fact that envelope coding changed with training suggests that there may also be a top-down central gain effect resulting from auditory deprivation (Munro and Blount, 2009). However, there was no change in TFS coding in the training group. The effects of hearing loss on envelope and TFS representation are a function of complex interactions among cochlear function, stimulus presentation level, and SNR (Henry and Heinz, 2012). One possibility is that a change in TFS coding was too subtle to be observed at the single SNR we used. Another important consideration is the nature of the training: all of the training stimuli were presented in quiet, not in noise. It is possible that this presentation technique favored envelope coding, which is maladaptively enhanced even in quiet in listeners with cochlear hearing loss (Anderson et al., 2013a). Further investigation to establish the effects of hearing loss at different presentation levels and SNRs is warranted, as a better understanding of the mechanisms underlying abnormal stimulus encoding will help guide future treatment efforts.

The results of our study contrast with those of two previous studies that found training-induced increases in envelope coding in normal-hearing young adults using different types of training: pitch discrimination training (Carcagno and Plack, 2011) and recognition of speech presented in babble and other challenging conditions (Song et al., 2012). The key difference between these studies and ours is that our study population included individuals with hearing impairment. Given the differences between normal-hearing and hearing-impaired listeners in performance on perceptual tasks, neurophysiological encoding of sound, and reliance on cognitive mechanisms for speech intelligibility (Lorenzi et al., 2006; Anderson et al., 2013a; Humes et al., 2013), it is not necessarily surprising that there are different effects of auditory training. Another important difference is the training itself. The training in our study directed attention to fast-changing sounds in high memory load situations and occurred in quiet. It may be that training on recognition of speech in noise, such as in the Song et al. study, produces more robust gains in speech-in-noise perception and different outcomes when comparing pre and post envelope and TFS coding. As another example, FFR neural representation of the F_0_ is correlated with behavioral pitch discrimination, (Krishnan et al., 2010; Marmel et al., 2013); therefore, enhanced neural representation of cues contributing to the perception of pitch may underlie the training-induced gains in pitch discrimination found by Carcagno and Plack (2011). Thus, outcomes may vary depending on the type of training and the targeted population.

We propose, therefore, that the training effects in this study were influenced by neural mechanisms specific to older adults with hearing loss. Both aging (Turner et al., 2005; Schatteman et al., 2008) and hearing loss (Vale and Sanes, 2002; Dong et al., 2009) appear to cause an imbalance in excitatory and inhibitory function, likely affecting stimulus encoding. However, this imbalance is at least partially reversed in animal models of auditory training (de Villers-Sidani et al., 2010) and acoustic experience (Turner et al., 2013). Although we are unable to verify these effects in humans, we speculate that the training-induced changes were facilitated by alterations in the balance of neurotransmitter levels to allow for precise encoding of subtle CV differences. That said, just as it is difficult to disambiguate between peripheral (i.e., outer hair cell loss and peripheral neuropathy) vs. central mechanisms of hearing loss, it is difficult to say with certainty which mechanisms were targeted by training.

Our training was designed to strengthen sensory function through attention to meaningful sound; specifically, focusing attention on CV transitions in speech may drive top-down modulation, which occurred in five of the six training modules. Animal models have demonstrated that directed attention to behaviorally-relevant stimuli is necessary for neural and behavioral plasticity (Fritz et al., 2005, 2007). In our study, we found that neural response changes were accompanied by improvements in attention. A functional connection between prefrontal and auditory cortices provides a basis for efferent activation during difficult tasks that require focused attention (Raizada and Poldrack, 2007). A tentative connection has also been suggested between prefrontal cortex and auditory brainstem based on fMRI studies (Roelfsema et al., 2010). Although our methodology does not allow us to draw similar conclusions, we propose that rapid TFS cues may become more salient when sensory and cognitive demands drive a prefrontal-brainstem connection to adjust subcortical encoding of these cues.

A number of questions remain unanswered. We did not include anyone in our study who wore hearing aids, because hearing aids themselves may induce plasticity in auditory processing (Munro et al., 2007; Munro and Merrett, 2013). Therefore, the use of amplification itself can be considered a form of training. Hornickel et al. (2012) demonstrated that assistive listening devices engender improved trial-to-trial consistency in brainstem firing, presumably by directing attention to a more robust and noise-free representation of the stimulus. Future work should compare the neural effects of amplification alone vs. amplification plus training. In addition, more work should be done to determine the persistence of training effects and the time course of learning. A number of approaches were employed in our training protocol, involving strictly bottom-up discrimination training and combined memory and perceptual training exercises. It would be important to identify the aspects of training that were primarily responsible for engendering plasticity. We note that there is a great deal of individual variability in the participants with hearing loss, both in terms of pre-training spectral amplitudes and in the degree of change. In future work, it will be important to identify sources of variability, and the factors that contribute to success in individuals.

Finally, although neither envelope nor TFS representation changed in the normal hearing group, this group did experience changes in attention and short-term memory. Therefore, we assume the normal-hearing participants experienced training-induced biological changes that were not observed using the current envelope/TFS technique. The lack of observed improvement in memory in the group with hearing loss may be due to the auditory nature of our assessment measure. Although the presentation level was adjusted to a comfortable volume for each participant, it may be that peripheral hearing loss limited performance, even in the post-training session, given the known effects of hearing loss on verbal short-term memory (McCoy et al., 2005; Verhaegen et al., 2013). The speech-in-noise perception scores (QuickSIN) of normal hearing groups would be considered clinically normal—less than 3 dB SNR loss (Killion et al., 2004), and so the lack of improvement in this group probably stems from a ceiling effect. Further investigation is needed to determine the mechanisms of training-induced improvements in individuals with normal hearing.

These results have implications for management of hearing loss problems in older adults. Although provision of audibility is a necessary foundation for the treatment of hearing difficulties, we assert that auditory training is just as important. The combination of amplification and auditory training may lead to improved speech clarity in noise without distortion from, overamplification.

### Conflict of interest statement

The authors declare that the research was conducted in the absence of any commercial or financial relationships that could be construed as a potential conflict of interest.
